# Protein Signatures to Trace Seafood Contamination and Processing

**DOI:** 10.3390/foods9121751

**Published:** 2020-11-26

**Authors:** Iciar Martinez, Isabel Sánchez-Alonso, Carmen Piñeiro, Mercedes Careche, Mónica Carrera

**Affiliations:** 1 Research Centre for Experimental Marine Biology & Biotechnology-Plentzia Marine Station (PiE), University of the Basque Country, UPV/EHU, Areatza 47, 48620 Plentzia, Spain; 2 IKERBASQUE Basque Foundation for Science, Euskadi Plaza, 5, 48009 Bilbao, Spain; 3 Department of Products, Institute of Food Science, Technology and Nutrition (ICTAN), Spanish National Research Council (CSIC), c/José Antonio Novais 10, 28040 Madrid, Spain; isabel.sanchez@csic.es (I.S.-A.); mcareche@ictan.csic.es (M.C.); 4 Scientific Instrumentation Department, Institute of Marine Research (IIM), Spanish National Research Council (CSIC), 36208 Vigo, Spain; cpineiro@iim.csic.es; 5 Food Technology Department, Institute of Marine Research (IIM), Spanish National Research Council (CSIC), 36208 Vigo, Spain; mcarrera@iim.csic.es

**Keywords:** seafood, traceability, safety, authentication, proteomics, FT Raman, FTIR, LF-NMR relaxometry, microplastics, drugs, medicines, frozen/thawed, time and temperature history

## Abstract

This review presents some applications of proteomics and selected spectroscopic methods to validate certain aspects of seafood traceability. After a general introduction to traceability and the initial applications of proteomics to authenticate traceability information, it addresses the application of proteomics to trace seafood exposure to some increasingly abundant emergent health hazards with the potential to indicate the geographic/environmental origin, such as microplastics, triclosan and human medicinal and recreational drugs. Thereafter, it shows the application of vibrational spectroscopy (Fourier-Transform Infrared Spectroscopy (FTIR) and Fourier-Transform Raman Spectroscopy (FT Raman)) and Low Field Nuclear Magnetic Resonance (LF-NMR) relaxometry to discriminate frozen fish from thawed fish and to estimate the time and temperature history of frozen fillets by monitoring protein modifications induced by processing and storage. The review concludes indicating near future trends in the application of these techniques to ensure seafood safety and traceability.

## 1. Introduction to Traceability and Safety

The General Food Law entered into force in the European Union in 2002 [1], making traceability compulsory for all food and feed businesses. It happened mainly as a response to the so-called “dioxin scandal” that took place in Belgium in January 1999, when polychlorinated biphenyls (PCBs) and dioxins were accidentally mixed with recycled fats that ended up being used in the production of animal feeds that were distributed to chicken and pig farms [2]. Over 30 countries and numerous food products were involved in the scandal, which had a major economic impact and originated an international crisis that challenged the ability of the authorities to ensure food safety. It also challenged the public perception of, and trust in, the food industry and international food trade [2,3].

Traceability is currently a key system for manufacturers to ensure the quick and efficient retrieval of damaged products if something goes amiss during the production chain. Hand in hand with product traceability should be its correct labelling, critical for the consumer to get truthful information and decide whether the product fulfils the expectations of identity, quality and safety and matches the price that the consumers are willing to pay. The price most often depends on the species declared, its geographic origin and processes to which it has been submitted [4]. Traceability misinformation and missing information may arise due to accidental loss of information, not collecting relevant information and/or the registration of wrongful information due to either honest mistakes or outright fraud. In those cases, the need arises for scientific analytical methods to verify the composition of the food matrix itself and, depending on the type of information required (identify the species, geographic origin, production, the processes that the product has gone though, its composition, safety and remaining shelf-life), one should select the most adequate diagnostic marker to provide the desired information (which are usually DNA/proteins, DNA/trace elements/contaminants, DNA/gene expression/protein regulation and protein/lipid structural modifications) [5,6,7].

**Figure 1:**
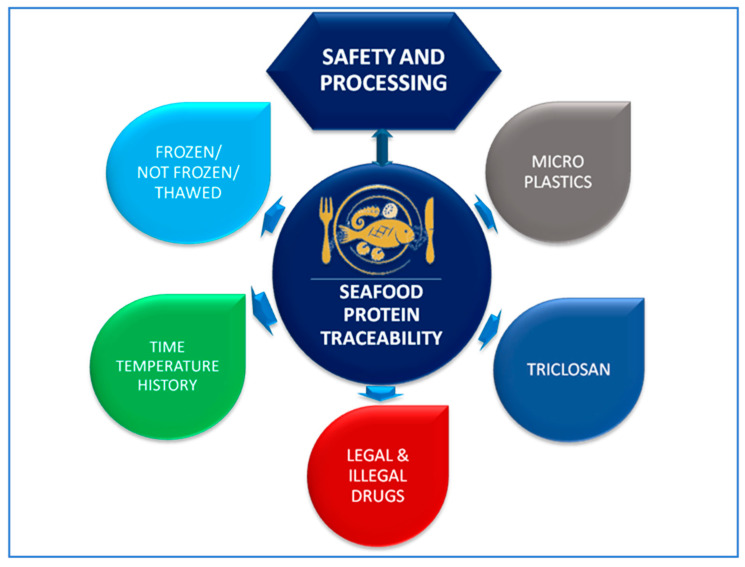
Selected traceability issues for which protein markers are reviewed in this work.

The present work will review the application of proteomics and spectroscopic techniques to identify protein markers in seafood suitable to address a number of traceability issues (shown in Figure 1) all along the production chain, that is, from the farm to the table. The scope will be limited, in the case of proteomics techniques, to the identification of exposure to emergent contaminants with implications for seafood safety and with the potential to identify the geographic origin. The latter is possible given the location-specific quantitative and qualitative distribution of contaminants, although no practical routine applications have been implemented yet. The selected applications of spectroscopic techniques include the verification of processing and storage conditions, which have practical applications both in the detection of fraud regarding frozen-thawed product labeling and to ensure its safety, when freezing is required by law to inactivate anisakid nematodes [8]. Table 1 lists some advantages and disadvantages of the selected techniques.

## 2. Proteomics: Discovery and Targeted Strategies

**Figure 2:**
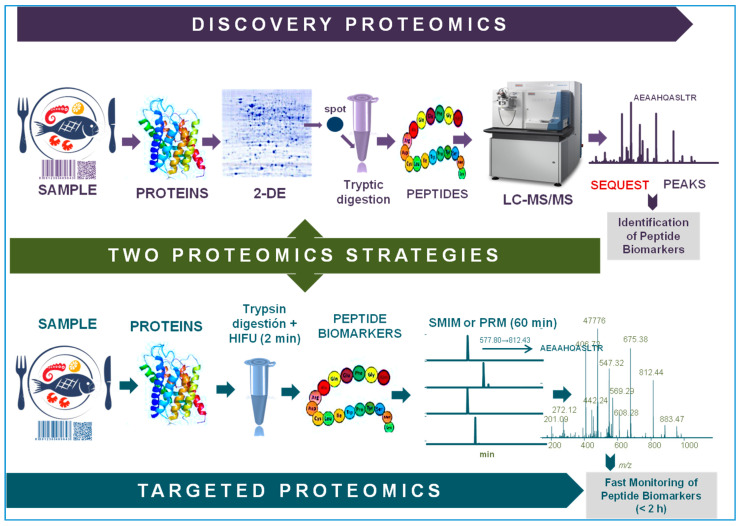
Schematic representation of discovery (**top**) and targeted (**bottom**) proteomics strategies.

Discovery proteomics is used to identify protein or peptide biomarkers in a given proteome, that is, proteins differentially regulated in control and experimental samples [9,10] (Figure 2). Bottom-up protocols can be divided into two different groups depending on whether the protein separation step is performed, or not, with the aid of a gel matrix. The classical method is to separate the proteins first by two dimensional gel electrophoresis (2DE) [9], identify diagnostic spots in the gel, excise and in-gel digest them into peptides, usually with trypsin, and identify the proteins by analyzing the resulting peptides by mass spectrometric (MS) techniques. Identification of proteins and their post-translation modifications, such as glycosylations [11], phosphorylations [12] and carbonylations [13], are performed using database search engines or by *de novo* MS sequencing [14]. In gel-free approaches, called shotgun proteomics, the entire sample is proteolyzed and the mixture of peptides is then analyzed by one or several (in tandem) liquid chromatographic techniques [15]. The eluted peptides are then fragmented and further processed by tandem mass spectrometry (MS/MS) [16,17]. The resulting spectra are assigned to putative peptide sequences using protein database searching algorithms like SEQUEST [18], Mascot [19], OMSSA [20] and X!Tandem [21] and the assignments are subsequently validated using additional software programs, such as PeptideProphet [22] and Percolator [23]. When the peptides are not contained in the databases [24], they must be *de novo* sequenced by interpreting the MS/MS spectrum either manually or by using dedicated programs such as Byonic [25], PEAKS [26] and UVnovo [27]. Protein quantification can be calculated from the intensity of the peptides produced by MS [28] or by methods which require prior peptide labeling. Common labeling methods include (i) isotope tagging using a chemical reaction, such as isobaric tags for relative and absolute quantitation (iTRAQ), tandem mass tags (TMTs) and difference gel electrophoresis (DIGE) [29,30,31]; (ii) incorporating a stable isotope through an enzymatic reaction (i.e., ^18^O) [32]; and (iii) labeling of metabolic stable isotopes (such as stable isotope labeling by/with amino acids in cell culture, SILAC) [33].

Top-down proteomics [34] does not require enzymatic digestion: intact proteins are applied to more sophisticated and accurate high-resolution MS instruments [35,36], where they are analyzed and fragmented.

Finally, the resulting peptides are compared to those contained in databases using alignment search programs (i.e., BLAST, https://blast.ncbi.nlm.nih.gov/) to identify the diagnostic peptide biomarkers. The identification of diagnostic markers is the main purpose of discovery proteomics. Targeted proteomics, on the other hand, monitors and detects those biomarkers and it is usually performed by MS (Figure 2) or by immunological techniques, that is, targeted proteomics focuses only on the identified specific markers for different relevant purposes (species identification, welfare indicators, presence of contaminants, etc.).

### 2.1. Classic Applications of Proteomics to Traceability

The proteome of an organism is determined by the species and its environment, that is, the interaction between nature and nurture. In the case of seafood, nature will be the species and stock of the organism and nurture would comprise all those external factors to which the organism is exposed to, such as physiological changes, exposure to stressors, drugs, medicines and contaminants, post-mortem elapsed time, storage temperature and conditions, processing methods and parameters. All those factors provide essential information to verify the traceability of seafood. The applications of proteomics techniques to traceability mentioned in this review are summarized in Table 2. The initial applications of 2DE proteomics to seafood traceability addressed the issues of species [37,38,39] and stock [39,40,41] identification. Recently, a proteome-wide MS/MS method performed following a simple, standardized protocol, that includes protein extraction, digestion and data analysis, has been successfully applied to heavily processed samples for authentication purposes [42]. Other initial applications of proteomics to traceability include the mapping of *postmortem* deterioration as a marker for freshness in shrimps [6], cod [43,44,45] and seabass [46]; and changes due to freezing and freezing storage in cod and rainbow trout [47,48,49]. These discovery-based studies identified, among others, parvalbumins, some myofibrillar proteins (myosin heavy and light chains, actin and α-actinin) and some enzymes (i.e., glyceraldehyde-3-phosphate dehydrogenases, nucleoside diphosphate kinase B and phosphoglycerate mutase 2) as good candidates for future targeted proteomics approaches to identify species and freshness [6,43,44,45,46]. Protein oxidation and carbonylation and changes in some enzymes involved in cellular metabolism and other functions (such as triose-phosphate isomerase, glyceraldehyde-3-phosphate dehydrogenase and aldolase A) were identified as markers for freezing and frozen storage [47,48,49]. All these markers have the potential to be used in targeted proteomics approaches.

The effect of processing on the proteome of different seafood products was examined in one of the earliest work identifying modifications in the 2DE map of surimi as a function of the *rigor* status of the cod [43], while a very recent work examined how high-pressure processing and enzymatic treatments act on the proteins of the shell of *Pandalus borealis* and thus contribute to the process of deshelling the shrimps [68].

From the early work in the 1990s and up to date, a wealth of excellent empirical work and reviews on food authentication by means of proteomics techniques have been published [36,50,51,52]. These studies address species authentication [7,53,54,55], different aspects of seafood quality, bioactivity and safety [56,57] and the production method (farmed vs. wild and health and welfare consideration) [57,58,59,60]. Similarly, the amount and composition of the feed taken by the fish prior to its death [60] (which affects its shelf life [81,82] and taste [83], respectively) and freshness and processing methods [51] have been examined by proteomics techniques. From the point of view of seafood safety, proteomics has been applied to identifying markers of exposure to traditional hazards, such as biotoxins [61,62] and environmental pollutants [63,64,65,66,67].

### 2.2. Application of Proteomics to Trace Exposure to Emergent Contaminants

The rest of this section will be devoted to the application of proteomics methodologies to identify seafood exposure to potential emerging hazards, such as microplastics [61,69], the personal care product triclosan [84,85] and pharmaceuticals and recreational drugs [86]. None of these contaminants have yet been reported to cause acute seafood intoxications, but given their prevalence and increasing amounts in the aquatic environment, they have the clear potential to become a public health issue. Furthermore, given that their presence and amount vary from location to location, they also have the potential to serve as markers for the geographic origin of seafood. We must keep in mind that the very incident that forced food traceability implementation had not been an issue of concern either, until it became a major one.

#### 2.2.1. Tracing Exposure to Microplastics

Microplastics have become ubiquitous in the aquatic environment [87] and even though their distribution has not been evenly mapped [88], they have been found in seafood [89], mostly in the gills and gut of the organisms, but also in the fillet of fish and the body of shrimps [90]. In the case of fish, gills and guts may be removed, but molluscs and some crustaceans are usually ingested whole.

The hazardous nature of microplastics is due to the chemicals used in their manufacture and the fact that they can absorb and retain in their surface organic and inorganic persistent, bioaccumulative and toxic contaminants, as well as potentially pathogenic microorganisms from the environment [88,89]. In laboratory experiments and at high concentrations, they have been shown to provoke physical and chemical toxicity causing stress, inflammation, blockage of the gastrointestinal tract and even physical injuries [88]. Although there is a lack of evidence indicating damage caused by conditions encountered in nature [88], we have nevertheless decided to include them in this review given that their presence as contaminants is only expected to increase in the future [89]. As of 2017, there were no analytical methods suitable to detect and quantify the smaller nanoplastics in the environment or in the food chain [89].

Sussarellu et al. [70] examined the effect of exposing reproductively active oysters (*Crassostrea gigas*) for two months to micro-sized polystyrene spheres (micro-PS, 2 and 6 μm, 0.023 mg/L), under controlled conditions suitable for germ cell maturation. Proteomic analysis was carried out by liquid chromatography-tandem mass spectrometry (LC-MS/MS) analyses of in-gel digested differentially expressed protein spots excised from 2DE gels. The microplastics, similar to phytoplankton, were efficiently ingested by the oysters, which consumed significantly larger amounts of microalgae and also increased their absorption efficiency, probably to compensate for the diminished food and energy intake caused by the micro-PS interfering in their digestive system. Transcriptomic analyses revealed differences in the expression of numerous genes. However, only two differentially regulated spots were identified in the proteome of oocytes: arginine kinase, which indirectly influences embryonic biosynthetic activities, and severin, an actin-binding protein necessary to regulate the completion of cell division. The former was present in lower levels and the latter was present in higher levels in the oocytes of exposed females [70].

The proteome of the hemolymph of blue mussels (*Mytilus edulis*) was also altered by the exposure to polyethylene (PE) microplastics and to the bioplastic polylactic acid (PLA) [69]. The peptide mixture resulting from the tryptic digestion of crude hemolymph was analyzed by means of a QExactive high-resolution mass spectrometer connected to a Dionex Ultimate 3000 RSLCnano chromatography system. Protein identification and label-free quantification and normalization of MS/MS spectra allowed the identification of 2745 peptides, corresponding to 216 proteins from *M. edulis*. Exposure to PE induced more drastic quantitative changes than exposure to PLA and it involved proteins implicated in vital processes, such as detoxification, metabolism, structural development and immune regulation. In particular, exposure to microplastics induced increases in some metabolic enzymes involved in glycolysis and in myticin, an antimicrobial peptide. Myosin showed the potential to act as a diagnostic marker of exposure to micromaterials, since its amount was reduced after exposure to micro-PE plastics [69]. The alterations induced by the biodegradable PLA add to the growing volume of data on the potential safety issues of these plastics on live organisms in spite of their condition of being biodegradable.

The proteomic response of copepods (*Tigriopus japonicus*), after two generations of exposure and one of recovery to concentrations of PS microplastics comparable to those found in the environment, was examined by Zhang et al. [71] who identified 4058 proteins after LC-MS/MS analysis. Exposure to microplastics elicited a reduction in energy storage and an increase in some cellular biosynthesis processes, which compromised survival and reproduction of the treated copepods. Two-generation exposure to microplastics upregulated several cellular processes, such as immune defense and protein biosynthesis. Upregulation affected proteins involved in translation, peptide biosynthetic process, gene expression, cytoplasmic and ribosomal enzymes, GTPase activity and C-type lectin-like/link domain [71].

In the freshwater zebra mussel (*Dreissena polymorpha*), exposure to mixtures of virgin PS microbeads of different sizes (1 and 10 µm diameter) altered the proteomic profile of the gills, but not at the lowest concentration used by the authors, suggesting a threshold concentration (higher than a mixture of 1 × 10 ^6^ microbeads/L) for the type of microplastics they used to exert their effect [72]. The proteins whose abundance was modulated were implicated in oxidative stress responses and they were mainly involved in energy metabolism, the structure and function of ribosomes, RNA binding and cytoskeleton and cellular trafficking [72]. Exposure to the higher dose of 4 × 10^6^ microbeads/L altered the amount of 78 different proteins and, strikingly, 18 of those proteins were absent in the zebra mussels exposed to the higher concentration of microplastics when compared with controls. The exposure induced diffuse effects on many protein classes, but no specific metabolic pathways were altered. In more detail, the major classes of proteins modified were those with catalytic and nucleotide-binding activities followed by proteins involved in protein binding, proteins related to RNA and proteins related to metal-ion-binding classes. Many of these proteins, which are directly or indirectly involved in the oxidative stress homeostasis, are mainly involved in energy metabolism, the structure and function of ribosomes, cellular trafficking, RNA binding and cytoskeleton and cellular trafficking [72], corroborating the results of the previously mentioned work [69] and the effect of microplastics on the increase of oxidative stress and the consequent imbalance of the antioxidant defense mechanisms. The exposure also induced an upregulation of ribosomal proteins and glutathione reductase and it modulated differently the expression of the cytoskeleton proteins: five were upregulated (actin-related protein, myosin-Ie, tubulin β chain, dynein arm light chain and light chain roadblock), two were downregulated (tropomyosin 1 and α-actinin) and four of them were not expressed (actin, β-actin, tubulin α-chain and septin-2) [72]. Exposure to microplastics also impacted on many proteins involved in RNA translation and protein synthesis. The role of these changes as potential indicators of defense mechanisms can be corroborated by the very low levels of some proteins. For example, three heat shock proteins (HSP cognate 70 kDa protein, HSP70 and putative HSP90) were below the detection limit, one was downregulated (HSP90 protein) and only one was upregulated (HSP cognate 70) compared with controls. The blockage of the expression of these HSPs may indicate that, being housekeeping proteins, their mRNA may remain untranslated for cellular energy-saving rather than playing a role as a possible barrier against injuries caused by the exposure to microplastics. Other energy pathways were also modulated by the exposure, an effect already noticed by Sussarellu et al. [70] in the oysters mentioned above in which the energy necessary for organism maintenance and growth was preserved at the expense of reproduction. The last big protein class modulated in zebra mussels by exposure to microplastics was related to protein degradation and included components of the proteasome, which is the structure involved in the degradation of ubiquitinated proteins [72].

#### 2.2.2. Proteomic Markers to Trace Exposure to Triclosan

Triclosan is a broad spectrum, non-antibiotic, antimicrobial agent that has become a persistent pollutant in soil, air and water, due to its use in over 2000 consumer products, such as toothpastes, deodorants, skin creams and soaps [91,92]. It has been found in drinking waters in concentrations up to 0.36 nM and in different aquatic systems, up to 7–10 nM in wastewater treatment plants, although 0.2–0.3 nM was more frequently recorded in streams with input of raw wastewater. More importantly, it has been found in concentrations up to 7.9 nM in surface waters of natural streams and rivers [91] and in marine sediments in concentrations of 9.3–450 nmol/Kg [92].

Due to its antimicrobial action, its potential to contribute to the development of bacterial resistance to antibiotics has been studied and proved [93,94], but triclosan is also an endocrine disruptor with a potential to bioaccumulate in fatty tissues and exert a toxic action on aquatic organisms [91]. It is also a cause for concern regarding human and environmental health since it has been detected in human samples of urine, nails, breast milk and blood, although there are no data on its bioaccumulation in humans. Triclosan is a pro-oxidant substance that may be cytotoxic, and whose estrogenicity and anti-estrogenicity may play a significant role in cancer progression, in addition to being considered as a potential causative agent for certain allergies and reproductive defects in humans based on epidemiological studies [91]. In addition, its presence in chlorinated drinking water may contribute to the formation of carcinogenic metabolites such as chlorophenols [91]. Therefore, we consider of relevance the development of analytical techniques to trace the exposure of seafood to this compound. We have not found any work studying proteomic changes induced by exposure to triclosan on commercially relevant species of marine seafood, but there are a few studies examining freshwater mussels and model fish.

Riva et al. [95] examined the effect of triclosan exposure on the proteome of the cytosolic extract of the gills of the freshwater mussel (*Dreissena polymorpha*) by 2DE and matrix assisted laser desorption/ionisation - time of flight/time of flight mass spectrometry (MALDI-TOF/TOF). A total of 12 proteins were found to be significantly altered by exposure to triclosan with marked effects on several biological processes, particularly those involved in stress responses and calcium binding. Peptide map fingerprinting and the MASCOT search engine allowed the tentative identification of seven proteins, all of which were upregulated: some were cytoskeletal (myosin light chain, tubulin α2/α4 chain and tubulin β4 chain) and others were involved in stress responses (HSP70, heat shock cognate 70 kDa protein and omega-crystallin) and in metabolism (glyceraldehyde-3-phosphate). MS/MS and the Global Proteome Machine software helped to tentatively identify two more proteins: the cytosolic malate dehydrogenase, which was downregulated, and a sarcoplasmic calcium-binding protein, which was upregulated.

A proteomic analysis using 2DE-DIGE workflow was used to study the effect of early exposure to triclosan on zebrafish larvae [96]. In this early stage, triclosan induced oxidative stress and neurotoxicity, in particular, affecting proteins involved in cytoskeleton, stress response, eyes and neuronal development. These results were corroborated by the enzymatic analysis, suggesting impairment in glutathione metabolism and acute neurotoxicity.

#### 2.2.3. Tracing Exposure to Medicines and Recreational Drugs by Proteomics

Over 600 pharmaceutical substances have been identified in the environment worldwide [97]. Recreational and prescription drugs and their metabolites are being found with increasing frequency, not only in sewage and coastal waters [98,99,100], but also in the edible tissues of species used as food [90,100]. The latest report of the European project ‘Perspectives on Drugs’ [99] contains data on the analysis of 68 cities in 23 European countries to explore the drug-taking habits of their inhabitants and shows that the loads of the different stimulant drugs detected in wastewater in 2019 have increased compared with previous years. Monitoring of this kind of contaminants is a priority target of the European Water Framework Directive [101] and the EU Marine Strategy Framework Directive [102], but most countries have not implemented methods for their detection and monitoring. These compounds are currently excluded from food and feed safety monitoring programs.

Many of these substances have psychotropic effects and have been shown to alter the behavior of fish in different manners, not always as expected when considering their effects on human behavior. In a study of European perch, for example, the fish ingested and bioaccumulated in their fillet oxazepam from water contaminated at levels similar to those encountered in surface waters receiving input of treated wastewater. These fish fed more often and showed reduced sociality and increased activity [100]. Similar results were obtained in mosquitofish exposed to field-realistic doses of the antidepressant fluoxetine [103]. Moreover, in real-life, fish are exposed to mixtures of these drugs whose compounded effects are unpredictable. Currently, we know that these chemicals are in the environment, where their amount and variety are increasing, that they accumulate in edible tissues of seafood and that they constitute a real hazard whose risk has not been mapped. Consequently, we consider that the development and implementation of methods to trace exposure of seafood to these compounds should be a priority to ensure seafood safety for the consumers.

Simmons and co-workers [84] employed an untargeted shotgun proteomics approach to identify alterations in the proteome of plasma proteins of goldfish exposed to the wastewater effluents of Cootes Paradise Marsh, an area of concern in Lake Ontario, Canada, with wastewater treatment plants which are able to remove solids, bacteria and nutrients, but unable to remove all chemical contaminants. Exposure to the plume (the moving body of contaminated water) influenced the amounts of over 250 molecules (proteins and metabolites) related to the synthesis of cyclic AMP, liver necrosis, the amount of intracellular calcium and the synthesis and accumulation of lipids. The expression of 36 proteins in the plasma of caged male goldfish differed significantly in at least one exposure location in the Cootes Paradise Marsh compared with the reference sites. However, there were 61 plasma proteins whose amounts were significantly different between wild goldfish captured in the marsh and the controls. Interestingly, the expression patterns of 26 proteins displayed a trend that seemed to keep a relationship to the distance along the plume from the wastewater treatment plants [84].

The exposure of Atlantic salmon (*Salmo salar*) parr during 5 days to concentrations based on real maximum levels detected in various European freshwater locations (see [104] and references therein) of the human medicinal drugs acetaminophen (also known as paracetamol, a very common analgesic, about 55 µg/L), atenolol (a beta blocker used to treat high blood pressure and heart-associated chest pain, about 11 µg/L) and carbamazepine (an anticonvulsant used mainly in the treatment of epilepsy and neuropathic pain, about 8 µg/L) induced notable changes in the proteome of their livers [104]. Thus, exposure to atenolol induced changes in the expression of seven proteins and exposure to carbamazepine affected the expression of 15 proteins. Some proteins, such as some enzymes involved in energy metabolism, were modulated by exposure to even low levels of all three contaminants. Some other altered proteins were involved in osmotic regulation (by exposure to paracetamol) and a heme oxygenase (by exposure to carbamazepine). The authors noted a lack of coherence between the results obtained by proteomics and transcriptomic analyses, indicating that the current proteomics protocols may detect changes only in the most abundant and soluble proteins, generating an incomplete and biased picture [104].

The exposure for 28 days to 1, 10 and 100 mg/L of carbamazepine modified the proteome of 10-month-old Chinese rare minnows (*Gobiocypris rarus*) in a gender-specific manner [105], influencing the differential regulation of 47 proteins in females and 22 in males. The cellular processes affected were identified by pathway analysis and included cell proliferation, differentiation and apoptosis and the respiratory chain, indicating impaired energy homeostasis. Remarkably, 15 of the differentially regulated proteins were associated with carcinogenicity. In this work, the results of transcriptomic analysis were consistent with that of the proteomics.

Diclofenac, a non-steroidal anti-inflammatory drug (sold under several brand names, including Voltaren) [106], induced changes in the proteome of plasma and kidney proteins of American catfish (*Rhamdia quelen*), after 14 days of treatment with three concentrations of the drug (0.2, 2.0 and 20.0 mg/L). The authors identified significant modifications in the expression of 20 proteins related to nitric oxide synthesis, leukocyte migration, the inflammatory process and the complement cascade, by a shotgun proteomic approach using LC-MS/MS. The exposure also induced a significant decrease in the expression of the class I major histocompatibility complex in plasma, leading to a suppression of the innate immune system of the fish.

Cocaine and its main metabolites, benzoylecgonine and ecgonine methyl ester, are commonly encountered in freshwaters worldwide at concentrations that can induce negative effects on aquatic organisms, but there are only a few studies on their effects. Parolini et al. [86] examined the effects on zebrafish (*Danio rerio*) embryos, at 96 h post-fertilization, of exposure to two concentrations similar to those found in naturally contaminated waters (0.3 and 1.0 mg/L) of the three compounds. Pooled embryos were analyzed by 2DE followed by in-gel digestion of diagnostic spots and identification by MALDI-TOF/TOF. Their proteome was found to be significantly modified by the contaminants and the proteins whose expression was modified belonged to different functional classes, including those involved in stress responses, lipid transport, energy and metabolism, eye constituents and cytoskeletal proteins, as well as vitellogenins and crystallins.

An analysis by 2DE and reversed-phase ultra performance liquid chromatography electrospray ionization linear ion trap-orbitrap-mass spectrometry (RP-UPLC ESI-LTQ-Orbitrap) of the gill proteome of freshwater mussels (*Dreissena polymorpha*) exposed during 14 days to two different concentrations (0.5 and 1 µg/L) of benzoylecgonine, indicated imbalances in oxidative stress and changes in proteins whose functions are critical to overall metabolism, including those involved in Ca^2+^ homeostasis [107].

## 3. Vibrational and LF-NMR Spectroscopy

Unlike proteomics techniques, spectroscopic analyses can be non-destructive and may require little sample preparation. They can also be highly specific, cost effective and fast to perform. At the same time, and similar to proteomic techniques, they can be used to measure several indicators simultaneously. The advantages and disadvantages and the applications of spectroscopic analyses to traceability aspects mentioned in this review are summarized in Table 1 and Table 2, respectively.

### 3.1. Vibrational Spectroscopy

Infrared (IR) and Raman spectroscopies are widely used techniques for quantitative and qualitative analyses of foods [108,109,110]. In a pioneering review on the applications of Raman spectroscopy in food science, Li-Chan [111] suggested the use of this technique as a tool for food control, for compositional identification or detection of adulteration and also for basic research studies to elucidate the structural changes occurring in food matrices as the consequence of processing. More recently, the applications of vibrational spectroscopy in food science have been collected in a book covering the instrumentation and fundamental applications as well as the analyses of food, drink and related materials [112,113]. The major constituents of fish muscle (proteins, lipids and water) can be detected by vibrational spectroscopy and the main structural features detected are related to secondary and tertiary protein structure, lipid oxidation and hydrolysis, lipid intramolecular and intermolecular order, water structure and water–biomolecule interactions [114].

These spectroscopies are based on the transitions between quantized vibrational energy states of molecules. The radiation in the IR region of the electromagnetic spectrum supplies the energy for those transitions in IR spectroscopy, whereas a source of monochromatic radiation (that may be in the near IR, visible or ultraviolet regions) is needed for the excitation of samples in Raman. In Raman spectroscopy, most of the incident photons return to the lowest vibrational ground state and only a small fraction of them return to an excited vibrational state, causing a shift in the energy of the scattered and incident photons that represent the energy of a vibrational transition within the sample molecule. Mid-IR and Raman spectroscopy can provide complementary information [108,109] since some vibrational motions, such as C=C and S-S polarizable groups and aromatic rings, have strong Raman bands, while O-H, N-H and C=O polar groups display strong stretching IR vibrations.

Regarding protein analysis, correlations between the IR and Raman spectra and protein secondary structure are well established and they can also be used to gather information about the local environments of protein side chains. Water molecular species, mostly corresponding to stretching (ν) and bending (δ) vibration bands, are also observed in different regions of a spectrum, while for muscle structural analyses the most useful water bands are those of ν (O-H) IR and Raman vibrations [115].

Raman spectroscopy has the advantage over IR spectroscopy that the samples require less manipulation, but the latter is less time consuming for spectral measurements. Raman spectral measurements of fish muscle can be carried out using sealed glass or quartz tubes of about 2–5 mm diameter, which should be thermostated to avoid sample alterations, for example, burning by the laser beam. The interference of fluorescence in Raman analysis can also be diminished by exciting with laser beams of a relatively long wavelength such as 785 nm or 1064 nm. IR analyses, on the other hand, can be performed by transmission or Attenuated Total Reflection (ATR). For the analysis of fish muscle by transmission infrared spectroscopy, a very small amount of sample (0.5–1 mg) is placed between two quartz windows and a slight pressure is applied between them in order to get a thin film of sample, free of bubbles and completely covering over the diameter of the infrared beam focus. In general, ATR is preferred since it does not require pretreatment of the sample, that is, a small amount of muscle is just placed in contact with a horizontal ATR accessory and the strong water absorption inherent to transmission infrared spectroscopy is significantly reduced in ATR.

### 3.2. Low-Field Nuclear Magnetic Resonance (LF-NMR) Relaxometry

LF-NMR relaxometry is an increasingly popular method for seafood analyses that has been applied to evaluate some characteristics of the raw material, such as muscle composition, handling prior to capture, *rigor mortis, postmortem* events and some processing conditions including salting, smoking and storage at above or below freezing temperatures [73,116,117,118]. LF-NMR studies the relaxation of protons placed in a static magnetic field after being exposed to a radiofrequency pulse. The transverse relaxation time (T_2_) is the most commonly monitored parameter in fish muscle. The decay curve obtained after relaxation is multi-exponential and, again in fish muscle, it is typically resolved into two, or more frequently three, components with their characteristic relaxation times. The shortest relaxation time (1–10 ms) is usually attributed to water tightly associated with macromolecules [117] or to non-exchangeable protein protons [119]. The components which have shown higher changes as a function of processing and/or storage conditions are the so-called T_21_ and T_22_, corresponding to intermediate and slow relaxing elements, respectively. Although dipolar interactions (i.e., proton interactions across space) leading to relaxation of water protons are modulated by rotation and diffusion, the chemical exchange is a factor that affects the modifications in the signal in protein systems with an excess of water. Thus, exchange between exchangeable protons from protein and water molecules leads to increasing apparent relaxation rates of water [119,120]. Therefore, despite the relative lower population of exchangeable protons from the proteins, since their relaxation rates are much higher than those of bulk water, the resultant observed relaxation may be heavily biased toward the relaxation of the protein [120] and changes in protein concentration, denaturation and aggregation will dictate modifications of the relaxation rate [120,121]. The exponential component (i.e., T_22_) is usually attributed to an increase in the heterogeneity in fish samples, which must exist on a large distance scale compared to the diffusion of water molecules so that water diffusion is not sufficiently fast and all water molecules experience all environments on a short time scale compared to the chemical exchange time scale [119]. LF-NMR relaxometry analysis is performed on small portions of samples (~2 g) placed into NMR glass tubes (18 mm diameter and 18 cm height) and the measurement takes less than 1 min, making the overall procedure very easy and fast to perform.

### 3.3. Application of Spectroscopic Techniques to Trace Protein Changes in Frozen Fish Muscle

Freezing followed by frozen storage is one of the most used and reliable methods for the long-term preservation of fish products, given that, in the frozen state, the rate of many chemical and physical reactions is substantially decreased and microbial growth is halted. There are many factors, such as fish species, season of capture, fishing/slaughtering methods, handling and storage of the fish prior to freezing, the freezing kinetics themselves, the frozen storage conditions and the interactions among all of the them, that may ultimately affect the characteristics of frozen products through modifications in the composition, structure and functionality of the components of the food matrix. Whereas the main changes caused by frozen storage are due to the development of rancidity in fatty fish, the key issue in lean species is the loss of a desirable texture: liquid loss upon thawing may occur and ultimately the products become dry, fibrous and hard. Semi-fatty species are usually the most stable toward frozen storage. All these changes may lead to a decreased acceptance by the consumer with the consequent loss of economic value.

Freeze-induced alterations include morphological changes such as modifications in the structure and ultrastructure of muscle fibers due to the pressure exerted by ice crystals. With slow freezing, extracellular ice crystals are formed, fibers are separated into groups and myofibrils become compressed, deformed and even fragmented. At high freezing rates, intracellular ice crystals can also provoke some fragmentation and certain looseness of myofibrils [122]. The final size of the ice crystals is a function of the freezing rate and final temperature [123]. In species such as cod and hake, the intermyofibrillar space is significantly reduced at high storage temperatures, whereas looseness is kept for a long time at low temperatures (i.e., −30 °C) [78,124]. Fish proteins may suffer denaturation and aggregation upon freezing and frozen storage due to the subsequent dehydration occurring by sublimation of ice crystals, the increased concentration of solutes in the unfrozen water portion of muscle and the formation of some compounds which may interact with the proteins such as formaldehyde in some species with high contents of trimethylamine oxide, free fatty acids from lipid hydrolysis and by-products from lipid oxidation [125,126,127].

Spectroscopic techniques can provide information of the structural changes described above. For example, alterations of the secondary protein structure have been shown by Raman spectroscopy and include a decrease of the native α-helices and the concomitant increase of β-sheet structures, which result in modifications in functionality and texture in a lean species such as hake [79,128]. The modifications of the Raman νCH band near 2935 cm^−1^ have been attributed to the exposure of aliphatic hydrophobic groups to the protein surface [128] and the changes in the intensity of the band near 160 cm^−1^ and the 3200 cm^−1^ νOH band have been attributed to changes in the distribution and mobility of water and protein–water interactions [78].

LF-NMR T_2_ relaxometry is also sensitive to different freezing and frozen storage conditions, which are known to affect ice crystal formation, alter fiber morphology and produce protein structural changes. Upon freezing, the following changes have been documented: the appearance of an extra component with a long relaxation time of frozen cod muscle [129], a significant increase in T_22_ in trout [130] and changes in T_21_ relaxation time and relative abundance in hake [131], saithe [132], salmon [133,134] and seabass after several freeze-thaw cycles [135]. Changes in these parameters were also found due to frozen storage in cod, salmon or hake [129,133,136,137,138,139].

**Figure 3:**
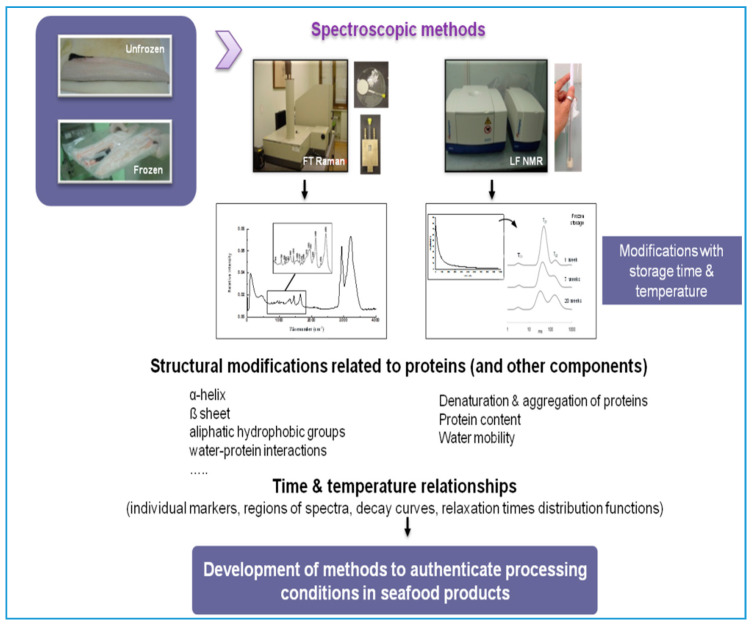
Application of spectroscopic methods (i.e., FT Raman, FTIR and LF-NMR) to authenticate frozen fish processing parameters.

Many of the protein changes described above and measured by spectroscopic techniques can be used alone as markers for freezing-induced modifications due to temperature abuse or prolonged storage time. However, not only proteins, but other components, may be altered by freezing and it is usually more convenient to use some selected regions or the whole spectral range containing as many as possible of the frozen storage-induced modifications, submit them to chemometric and classification techniques and/or use them to produce kinetic models (Figure 3). The ultimate purpose is to trace the actual time–temperature history of the product to verify the one provided by the traceability paperwork and to estimate the remaining shelf-life of the product.

#### 3.3.1. Time–Temperature History of Frozen Fish

One of the approaches to estimate the time–temperature history of frozen fish consists in using the well-established relationship between the storage temperature and the rate of physical–chemical reactions occurring in frozen stored fish muscle given by the Arrhenius equation. This approach has been used to estimate the shelf-life of a number of food systems [140,141], since it allows to estimate the temperature when the storage time is known and *vice versa*. Both parameters should be provided by the traceability information and this approach can be used to verify their reliability. Both vibrational spectroscopy and LF-NMR relaxometry can provide markers suited for that purpose [73]. In a series of work, it was found that Arrhenius models can be obtained by using the Principal Component (PC) scores of the spectroscopic outputs as response variables to model the time–temperature relationships. Here, we explain this approach for LF-NMR relaxometry data [74]. In order to estimate the suitability of the spectroscopic methods for that purpose, the evolution of the signals with frozen storage time at a fixed temperature was explored first, followed by the examination of the possible changes at selected temperatures in a relatively short storage time. Initial observations showed that T_2_ relaxation times (T_21_ and T_22_) at a fixed temperature provided information that was (i) correlated with storage time and quality-related parameters, such as shear resistance and water-holding capacity [139] and (ii) related to changes in the fillet as affected by freezing rate, temperature (−10 and −20 °C) and storage time (up to 12 weeks) [131].

Since practical storage time of a lean fish such as hake may be well beyond two years depending on the storage temperature, hake (*Merluccius merluccius* L.) fillets were stored at −10, −20, −30 or −80 °C for up to 150 weeks in order to investigate the potential of LF-NMR to produce indicators for the estimation of the quality and shelf-life of the frozen product by kinetic modeling. A factor analysis was performed using the mean data values of the T_2_ relaxation time distribution function, averaged by week, both in the range of 12 and 700 ms, and also separated in different ranges (12–112, 12–400, 12–700, 112–400 and 112–700 ms). The first PC of each of the analyzed ranges explained between 60 and 85% of the variance, and since all these PCs kept a relationship with temperature and time, they were used for kinetic modeling. In all cases, the highest R^2^ values were found for zero-order kinetics. The best models in terms of lower standard errors and higher R^2^ were obtained when using ranges where both T_21_ and T_22_ signals were included (12–400 and 12–700 ms). Although the differences were small, the best one was between 12 and 400 ms. The final kinetic model was performed in the selected range with all the data values and a good agreement was observed between the predicted and observed values, particularly at −10 and −20 °C where most of the changes were taking place [74,142]. Thus, the PC scores obtained from the first PC of the LF-NMR distribution function of T_2_ relaxation times followed the Arrhenius-type equation.

Along with this indicator, the time/temperature evolution of well-established physical and chemical markers for the species of interest would have to be compared with the spectroscopic data, either directly or through their corresponding kinetic parameters. Since this information was not found in the literature, markers such as water-holding capacity, shear resistance and phospholipid hydrolysis were subjected to kinetic modeling since they are, in turn, markers for sensory properties of the fillet, such as texture and taste [78,127]. The temperature dependency of the reaction rates thus estimated was similar to that of the shear resistance of the fillet, as was the hydrolysis of phospholipids after modeling the first PC scores of the FTIR lipid spectra [143,144]. The method based on LF-NMR is less informative in terms of precise protein structural changes but, as previously mentioned, it provides faster results given the possibility to perform the measurements directly on untreated samples.

In summary, these models can be useful tools to identify the exposure of batches of frozen fish to higher-than-reported temperatures along the distribution chain, thus becoming suitable authentication tools in cases of mislabeling or fraud.

#### 3.3.2. Discrimination of Fresh and Frozen–Thawed Fish

IR or Raman vibrational spectroscopy [75,79,145] and LF-NMR [131] have the potential to provide markers to discriminate unfrozen from frozen–thawed fillet, making them very useful tools to expose one of the most common frauds, namely the sale of thawed fish as “fresh”. Karoui et al. [75,76] applied PC and factorial discriminant analyses on three spectral ranges of the mid-infrared region. These were: (i) 1500–900 cm^−1^, corresponding to the so-called fingerprint region which refers to C-O and C-C stretching modes and O-C-H, C-C-H and C-O-H bending modes; (ii) 1700–1500 cm^−1^, which refers to the amide I and II bands, which give information on the proteins and interactions with other components such as water, ions and other proteins; and (iii) 3000–2800 cm^−1^, which corresponds to C-H modes from methyl and methylene groups of fatty acids [75]. The changes observed between the fresh and frozen–thawed fish samples in the 1500–900 cm^−1^ region were partially attributed to the difference in the level of water content, which was explained by protein denaturation, leading to decreased water-holding capacity of these proteins, which in turn was due to the tissue rupture caused by ice crystal formation and leakage of various organelles [75]. The 3000–2800 cm^−1^ region (dominated by two strong bands at 2920 and 2850 cm^−1^ assigned to the methylene anti-symmetric and symmetric stretching modes, respectively) showed different shapes between frozen–thawed and fresh fish samples, whereas no particularly informative modifications were observed in the 1700–1500 cm^−1^ region. The authors concluded that the 1500–900 cm^−1^ and particularly, the 3000-2800 cm^−1^ spectral regions were informative in order to discriminate between fresh and frozen–thawed samples, with 100% and 75% correct classifications for the calibration and validation models, respectively, for the 1500–900 cm^−1^ region and 100% and 87.5% correct classifications for the calibration and validation models, respectively, for the 3000–2800 cm^−1^ region.

Low-field NMR T_2_ transverse relaxation measurements were performed on hake (*Merluccius merluccius* L.) fillets first stored in ice for 3 and 14 days and then subjected to different freezing methods (air blast, liquid nitrogen or walk-in freezer) and storage conditions (−20 and −10 °C for 5 days, 8 weeks and 18 weeks). A major band (T_21_), centered between 47 and 60 ms, which accounted for 90–92% of the total signal, was found in unfrozen muscle. Upon freezing, T_21_ became wider and an extra band appeared within the range of 120–360 ms. Whereas no changes were detected at −20 °C, the T_21_ time constant decreased during frozen storage at −10 °C in a similar way for all three freezing methods. The relative abundance of T_21_ declined with storage time, but differences were found as a function of freezing. These changes were attributed to morphological alterations and protein denaturation [131]. A discriminant analysis rendered very successful classifications of fresh and frozen–thawed hake fillets (97% and 98%, respectively), indicating, again, the potential of these methodologies for fraud detection in the frozen-seafood chain [77].

Although the most common deception is to sell, as fresh, fish that has been frozen–thawed, there is an instance when fish must have been frozen to “at least −20 °C in all parts of the product for not less than 24 h, or −35 °C for not less than 15 h” [8] for safety reasons, namely, to inactivate the third larval stage (L3) of the parasite *Anisakis* that may be infecting the fillet [146]. However, given the higher perceived sensory quality of the unfrozen fillet, unfrozen fish may be offered to be consumed raw, endangering the health of the consumer.

In practice, the obligatory time-temperature conditions can be attained with a wide range of freezing kinetics that would in turn modify the characteristics of fish muscle to different degrees, in particular, when slow rates or high storage temperatures are applied. To be able to differentiate between legal (frozen–thawed) and fraudulent (not frozen) fish is more difficult than to differentiate between not frozen and thawed fish because of the short frozen storage time required by the legislation. Fortunately, LF-NMR relaxometry has also been shown to be suitable in this case: an analysis of fish treated at different time/temperature rate conditions known to inactivate *Anisakis* parasites provided data to develop a model that could discriminate between fish that had been frozen and fish that had not been frozen [80]. Thus, an additional application of vibrational spectroscopy and/or LF-NMR relaxometry may provide discriminant models able to verify the fulfillment of this EU Regulation [8].

## 4. Conclusions and Future Trends

Scientists must be ahead of the demands from the governments to the industry and prepare the tools necessary to ensure consumer safety and well-being. One such instance involves the validation of traceability information to detect the introduction of dangerous hazards in the seafood chain and to follow their distribution. Emergent hazards must be identified, their impact and effects mapped and biomarkers to identify both their presence in seafood and the exposure of seafood to them must be made available for practical screening of relevant foods. Proteomics hasa shown a clear potential to become a powerful tool to trace exposure to the emergent contaminants examined by these review: exposure to microplastics, triclosan and human medicinal and recreational drugs at concentrations naturally found in some environments induce significant alterations in the proteome of all tissues examined, including gills, plasma, liver, eggs and fillet. The most often influenced proteins are those related to stress responses, the cytoskeleton, enzymes involved in energy metabolism, protein and lipid synthesis and metabolism, heat shock proteins, Ca^2+^ and energy homeostasis and the immune system and, in some instances, proteins associated with carcinogenicity. Future developments will require the combination of proteomics methods and chemometrics to develop mathematical models for classification of samples according to their identity, processing and exposure to different hazards. One clear advantage of proteomics techniques is that once the discovery phase is completed, targeted strategies can detect the selected protein markers in a fast and efficient manner to provide relevant information on different traceability aspects such as species authentication, processing methods, geographic origin and wholesomeness.

Spectroscopic techniques have the potential to detect modifications in the protein structure due to processing in terms of protein denaturation and aggregation, which relate to important aspects of quality and safety that need to be preserved and traced (for instance, freezing and frozen storage abuse and/or fraud involving fresh vs. frozen/thawed or frozen labels). The applications described here by means of FT Raman, FTIR or LF-NMR relaxometry allow to combine the basic knowledge on protein changes and their interactions with other components, that is, selected wavelengths for FT Raman or FTIR or relaxation rates in LF-NMR with the use of chemometrics, where some parts or the whole spectra are being analyzed to differentiate or discriminate processing parameters. We have shown examples of models that may be applied to monitor the temperature–time storage conditions of fish lots and in this manner serve as an authentication tool when fraud or mislabeling occurs.

Ideally, the marker, or set of markers, should be specific to verify the information that needs to be authenticated but, perhaps except for genetically determined markers, that is rarely the case. For that reason, there is a need to carry out large-scale projects with many laboratories involved, in order to develop standard operation procedures to perform the analyses on well-identified reference and test samples covering the spectra of conditions (species, feeds, breeding, environmental conditions, exposure to contaminants, processing and storage parameters, etc.) that will need to be examined in real-life situations, build up databases and validate the methodologies by interlaboratory studies, so that they can finally become international reference methods.

## Figures and Tables

**Table 1:** Summary of advantages and disadvantages of proteomics and spectroscopic techniques.

Technique	Advantages	Disadvantages
***Proteomics techniques***		
Discovery proteomics	Protein spots are visible. Hundreds to thousands of polypeptides can be separated and analyzed in a single gel. Relative spot quantification is easy. Protein quantification is possible using differential labeling (e.g., fluorophores). Suitable to identify post-translational modifications. It is the most relevant method for the separation and *de novo* MS sequencing of incompletely sequenced organisms.	Expensive equipment. Manual work and high skills required. Time consuming (days). Gel-based approach allows the visualization of a relatively low number of proteins (<1000). Problems with the separation of poorly soluble, hydrophobic proteins and very low or very high relative molecular mass Mr proteins. Salt ions may interfere with protein separation. Suppression of signal by highly abundant proteins. Limited reproducibility. Smaller dynamic range than other separation methods.
Proteome wide MS/MS	Identification and quantification of thousands of proteins (>10,000) in a single experiment. This approach presents high sensitivity and reproducibility. It is automated. High-throughput experiments.	Loss of information on protein isoforms. It is difficult to identify proteins from species with unsequenced genomes. Manual work is required for unsequenced genomes. MS identifies only peptides: protein identification is indirect by comparison with the peptides originated from known proteins contained in databases. No match with experimentally measured Mr and pH.
Targeted MS/MS	High sensitivity and reproducibility. Automation is possible.	Low protein identification rate (<10%).
***Spectroscopic techniques***		
Spectroscopic techniques (in general)	Little or no sample preparation. They may be applied in a non-destructive or non-invasive way (i.e., in whole muscle). Low analysis cost. Short analysis time (seconds to minutes). Low amount of sample required. No need of high technical skills. Suitable for routine analysis. There is equipment for field analysis (i.e., suitable for laboratory, in situ and industry routine analyses). Alternative to wet chemistry.	No distinction among proteins in complex samples. Low sensitivity. Sometimes, initial high cost in equipment. Spectral data pre-treatment may be needed prior to data analysis. Portable instruments available only for selected applications.
Infrared and Raman spectroscopy	Highly specific. IR and Raman spectroscopy are complementary to each other. Minimum interference from water (Raman). Attenuated total reflectance (ATR) measurement mode overcomes the problem of strong water absorption in IR. Several indicators may be analyzed simultaneously. They provide analytical, structural, qualitative and quantitative information. Well-known secondary structure-spectral correlations. Libraries for compounds are available.	It requires skilled personnel. Fluorescence can hide the spectrum (Raman). Strong water absorption may be a problem (IR). Spectral data analysis is complex. The spatial variability of the sample may not be correctly taken into account.
Low-field NMR	Good relation with water-holding capacity and texture attributes.	Difficult interpretation in some scenarios.

**Table 2:** Summary of the applications of the analytical techniques cited in this review.

Technique	Application	Species	References
***Discovery proteomics***			
	Authentication	Numerous	[7,36,42,50,51,52,53,54,55]
	Species and stock identification	Numerous	[37,38,39,40,41]
	Quality, bioactivity and safety	Numerous	[56,57]
	Production method (wild vs. farmed)	Numerous	[54,57,58,59,60]
	Exposure to biotoxins	Bivalves, fish species	[61,62]
	Exposure to environmental pollutants	Ayu, rainbow trout	[63,64,65,66,67]
	*Postmortem* changes	Shrimp, cod, seabass	[6,43,44,45,46]
	Freezing and frozen storage	Cod, rainbow trout	[47,48,49]
	Surimi manufacture and *rigor* status	Cod	[43]
	High pressure and enzymatic deshelling	Shrimp	[68]
	Exposure to microplastics	Oysters, blue mussels, copepods, zebra mussel	[69,70,71,72]
***Spectroscopic techniques***			
IR ^1^, Raman ^2^	Time–temperature history of frozen fish	European hake	[73]
LF-NMR	Time–temperature history of frozen fish	European hake	[73,74]
IR ^1^	Discrimination fresh/frozen–thawed	European hake, several	[75,76,77]
Raman ^2^	Discrimination fresh/frozen–thawed	European hake	[78,79]
LF-NMR	Discrimination fresh/frozen–thawed	European hake	[80]

^1^ infrared spectroscopy, ^2^ Raman spectroscopy.

## References

[1] EU Commission (2002). Regulation (EC) No 178/2002 of 28 January 2002 laying down the general principles and requirements of food law, establishing the European Food Safety Authority and laying down procedures in matters of food safety. Off. J. Eur. Communities.

[2] Covaci A., Voorspoels S., Schepens P., Jorens P., Blust R., Neels H. (2008). The Belgian PCB/dioxin crisis-8 years later. An overview. Environ. Toxicol. Pharmacol..

[3] Buzby J.C., Chandran R. (2003). The Belgian Dioxin Crisis and Its Effects on Agricultural Production and Exports.

[4] Gracia A., De-Magistris T. (2016). Consumer preferences for food labeling: What ranks first?. Food Control.

[5] Martinez I., Aursand M., Erikson U., Singstad T.E., Veliyulin E., Van Der Zwaag C. (2003). Destructive and non-destructive analytical techniques for authentication and composition analyses of foodstuffs. Trends Food Sci. Technol..

[6] Martinez I., Friis T.J., Careche M. (2001). *Post mortem* muscle protein degradation during ice-storage of Arctic (*Pandalus borealis*) and tropical (*Penaeus japonicus* and *Penaeus monodon*) shrimps: A comparative electrophoretic and immunological study. J. Sci. Food Agric..

[7] Martinez I., Friis T.J. (2004). Application of proteome analysis to seafood authentication. Proteomics.

[8] EU Commission (2005). Regulation (EC). No 2074/2005 of 5 December 2005 laying down implementing measures for certain products under Regulation (EC). No 853/2004 of the European Parliament and of the Council and for the organisation of official controls under Regulatio. Off. J. Eur. Communities.

[9] O’Farrell P.H. (1975). High Resolution two-dimensional electrophoresis of Proteins. J. Biol. Chem..

[10] Pandey A., Mann M. (2000). Proteomics to study genes and genomes. Nature.

[11] Geng F., Huang X., Majumder K., Zhu Z., Cai Z., Ma M. (2015). Mass spectrometry and two-dimensional electrophoresis to characterize the glycosylation of hen egg white ovomacroglobulin. J. Agric. Food Chem..

[12] Mayer K., Albrecht S., Schaller A. (2015). Targeted analysis of protein phosphorylation by 2D electrophoresis. Methods Mol. Biol..

[13] Pazos M., Da Rocha A.P., Roepstorff P., Rogowska-Wrzesinska A. (2011). Fish proteins as targets of ferrous-catalyzed oxidation: Identification of protein carbonyls by fluorescent labeling on two-dimensional gels and MALDI-TOF/TOF mass spectrometry. J. Agric. Food Chem..

[14] Carrera M., Cañas B., Piñeiro C., Vázquez J., Gallardo J.M. (2007). *De novo* mass spectrometry sequencing and characterization of species-specific peptides from nucleoside diphosphate kinase B for the classification of commercial fish species belonging to the family merlucciidae. J. Proteome Res..

[15] Zhang Y., Fonslow B.R., Shan B., Baek M.C., Yates J.R. (2013). Protein analysis by shotgun/bottom-up proteomics. Chem. Rev..

[16] Wolters D.A., Washburn M.P., Yates J.R. (2001). An automated multidimensional protein identification technology for shotgun proteomics. Anal. Chem..

[17] Carrera M., Ezquerra-Brauer J.M., Aubourg S.P. (2020). Characterization of the jumbo squid (*Dosidicus gigas)* skin by-product by shotgun proteomics and protein-based bioinformatics. Mar. Drugs.

[18] Eng J.K., McCormack A.L., Yates J.R. (1994). An approach to correlate tandem mass spectral data of peptides with amino acid sequences in a protein database. J. Am. Soc. Mass Spectrom..

[19] Perkins D.N., Pappin D.J.C., Creasy D.M., Cottrell J.S. (1999). Probability-based protein identification by searching sequence databases using mass spectrometry data. Electrophoresis.

[20] Geer L.Y., Markey S.P., Kowalak J.A., Wagner L., Xu M., Maynard D.M., Yang X., Shi W., Bryant S.H. (2004). Open mass spectrometry search algorithm. J. Proteome Res..

[21] Craig R., Beavis R.C. (2004). TANDEM: Matching proteins with tandem mass spectra. Bioinformatics.

[22] Keller A., Nesvizhskii A.I., Kolker E., Aebersold R. (2002). Empirical statistical model to estimate the accuracy of peptide identifications made by MS/MS and database search. Anal. Chem..

[23] Käll L., Canterbury J.D., Weston J., Noble W.S., MacCoss M.J. (2007). Semi-supervised learning for peptide identification from shotgun proteomics datasets. Nat. Methods.

[24] Shevchenko A., Wilm M., Mann M. (1997). Peptide sequencing by mass spectrometry for homology searches and cloning of genes. J. Protein Chem..

[25] Bern M., Kil Y.J., Becker C. (2012). Byonic: Advanced peptide and protein identification software. Curr. Protoc. Bioinform..

[26] Ma B., Zhang K., Hendrie C., Liang C., Li M., Doherty-Kirby A., Lajoie G. (2003). PEAKS: Powerful software for peptide *de novo* sequencing by tandem mass spectrometry. Rapid Commun. Mass Spectrom..

[27] Robotham S.A., Horton A.P., Cannon J.R., Cotham V.C., Marcotte E.M., Brodbelt J.S. (2016). UVnovo: A *de novo* sequencing algorithm using single series of fragment ions via chromophore tagging and 351 nm ultraviolet photodissociation mass spectrometry. Anal. Chem..

[28] Mueller L.N., Rinner O., Schmidt A., Letarte S., Bodenmiller B., Brusniak M.Y., Vitek O., Aebersold R., Müller M. (2007). SuperHirn—A novel tool for high resolution LC-MS-based peptide/protein profiling. Proteomics.

[29] Mateos J., Landeira-Abia A., Fafián-Labora J.A., Fernández-Pernas P., Lesende-Rodríguez I., Fernández-Puente P., Fernández-Moreno M., Delmiro A., Martín M.A., Blanco F.J. (2015). iTRAQ-based analysis of progerin expression reveals mitochondrial dysfunction, reactive oxygen species accumulation and altered proteostasis. Stem Cell Res. Ther..

[30] Robotti E., Marengo E. (2018). 2D-DIGE and fluorescence image analysis. Methods Mol. Biol..

[31] Stryiński R., Mateos J., Pascual S., González Á.F., Gallardo J.M., Łopieńska-Biernat E., Medina I., Carrera M. (2019). Proteome profiling of L3 and L4 *Anisakis simplex* development stages by TMT-based quantitative proteomics. J. Proteom..

[32] López-Ferrer D., Ramos-Fernández A., Martínez-Bartolomé S., García-Ruiz P., Vázquez J. (2006). Quantitative proteomics using ^16^O/^18^O labeling and linear ion trap mass spectrometry. Proteomics.

[33] Ong S.E., Blagoev B., Kratchmarova I., Kristensen D.B., Steen H., Pandey A., Mann M. (2002). Stable isotope labeling by amino acids in cell culture, SILAC, as a simple and accurate approach to expression proteomics. Mol. Cell. Proteom..

[34] Fornelli L., Toby T.K., Schachner L.F., Doubleday P.F., Srzenti K., Dehart C.J., Kelleher N.L. (2018). Top-down proteomics: Where we are, where we are going?. J. Proteom..

[35] Carrera M., Cañas B., Vázquez J., Gallardo J.M. (2010). Extensive *de novo* sequencing of new parvalbumin isoforms using a novel combination of bottom-up proteomics, accurate molecular mass measurement by FTICR-MS, and selected MS/MS ion monitoring. J. Proteome Res..

[36] Carrera M., Eguiraun H., Cañas B., Martinez I., Piñeiro C., Caballero B., Finglas P.M., Toldra F. (2015). Proteomics: Contribution of proteomics techniques to understanding the interrelationship between food and health. Encyclopedia of Food and Health.

[37] Martinez I., Ofstad R., Olsen R.L. (1990). Myosin isoforms in red and white muscles of some marine teleost fishes. J. Muscle Res. Cell Motil..

[38] Ochiai Y., Kobayashi T., Watabe S., Hashimoto K. (1990). Mapping of fish myosin light chains by two-dimensional gel electrophoresis. Comp. Biochem. Physiol. Part B Comp. Biochem..

[39] Martinez I., Christiansen J.S. (1994). Myofibrillar proteins in developing white muscle of the Arctic charr, *Salvelinus alpinus* (L.). Comp. Biochem. Physiol. Part B Biochem..

[40] Martinez I., Ofstad R., Olsen R.L. (1990). Intraspecific myosin light chain polymorphism in the white muscle of herring (*Clupea harengus harengus*, L.). FEBS Lett..

[41] Martinez I., Bang B., Hatlen B., Blix P. (1993). Myofibrillar proteins in skeletal muscles of parr, smolt and adult atlantic salmon (*Salmo salar* L.). Comparison with another salmonid, the arctic charr *Salvelinus alpinus* (L.). Comp. Biochem. Physiol. Part B Comp. Biochem..

[42] Wulff T., Nielsen M.E., Deelder A.M., Jessen F., Palmblad M. (2013). Authentication of fish products by large-scale comparison of tandem mass spectra. J. Proteome Res..

[43] Martinez I., Solberg C., Lauritzsen C., Ofstad R. (1992). Two-dimensional electrophoretic analyses of cod (*Gadus morhua*, L.) whole muscle proteins, water soluble fraction and surimi. Effect of the addition of CaCl_2_ and MgCl_2_ during the washing procedure. Appl. Theor. Electrophor..

[44] Kjærsgård I.V.H., Jessen F. (2003). Proteome analysis elucidating *post-mortem* changes in cod (*Gadus morhua*) muscle proteins. J. Agric. Food Chem..

[45] Kjærsgård I.V.H., Nørrelykke M.R., Jessen F. (2006). Changes in cod muscle proteins during frozen storage revealed by proteome analysis and multivariate data analysis. Proteomics.

[46] Terova G., Addis M.F., Preziosa E., Pisanu S., Pagnozzi D., Biosa G., Gornati R., Bernardini G., Roggio T., Saroglia M. (2011). Effects of *postmortem* storage temperature on sea bass (*Dicentrarchus labrax*) muscle protein degradation: Analysis by 2-D DIGE and MS. Proteomics.

[47] Kjærsgård I.V.H., Nørrelykke M.R., Baron C.P., Jessen F. (2006). Identification of carbonylated protein in frozen rainbow trout (*Oncorhynchus mykiss*) fillets and development of protein oxidation during frozen storage. J. Agric. Food Chem..

[48] Jessen F., Lametsch R., Bendixen E., Kjærsgård I.V.H., Jørgensen B.M. (2002). Extracting information from two-dimensional electrophoresis gels by partial least squares regression. Proteomics.

[49] Kjærsgård I.V.H., Jessen F. (2004). Two-dimensional gel electrophoresis detection of protein oxidation in fresh and tainted rainbow trout muscle. J. Agric. Food Chem..

[50] Campos A.M.O., de Almeida A.M. (2016). Top-down proteomics and farm animal and aquatic sciences. Proteomes.

[51] Ortea I., O’Connor G., Maquet A. (2016). Review on proteomics for food authentication. J. Proteom..

[52] Piñeiro C., Carrera M., Cañas B., Lekube X., Martinez I., Nollet L.M., Toldrá F. (2015). Proteomics and food analysis: Principles, techniques, and applications. Handbook of Food Analysis.

[53] Rodríguez E.M., Ortea I., Colgrave M.L. (2017). Food authentication of seafood species. Proteomics in Food Science.

[54] Carrera M., Cañas B., Gallardo J.M., Colgrave M.L. (2017). Proteomic identification of commercial fish species. Proteomics in Food Science.

[55] Martinez I., James F., Loréal H. (2005). Application of Modern Analytical Techniques to Ensure Seafood Safety and Authenticity.

[56] Méndez L., Pazos M., Colgrave M.L. (2017). Chapter 18—Proteomics to assess fish quality and bioactivity. Proteomics in Food Science.

[57] Rodrigues P.M., Campos A., Kuruvilla J., Schrama D., Cristobal S., Colgrave M.L. (2017). Proteomics in aquaculture: Quality and safety. Proteomics in Food Science.

[58] Vaibhav V., Thompson E., Raftos D., Haynes P.A., Colgrave M.L. (2017). Proteomic analysis of disease in Sydney rock oysters. Proteomics in Food Science.

[59] Rodrigues P.M., Silva T.S., Dias J., Jessen F. (2012). Proteomics in aquaculture: Applications and trends. J. Proteom..

[60] Carrera M., Piñeiro C., Martinez I. (2020). Proteomic strategies applied to farming conditions in aquaculture. Foods.

[61] Fernández Robledo J.A., Yadavalli R., Allam B., Pales Espinosa E., Gerdol M., Greco S., Stevick R.J., Gómez-Chiarri M., Zhang Y., Heil C.A. (2019). From the raw bar to the bench: Bivalves as models for human health. Dev. Comp. Immunol..

[62] Karim M., Puiseux-Dao S., Edery M. (2011). Toxins and stress in fish: Proteomic analyses and response network. Toxicon.

[63] Lu X.J., Chen J., Huang Z.A., Zhuang L., Peng L.Z., Shi Y.H. (2012). Influence of acute cadmium exposure on the liver proteome of a teleost fish, ayu (*Plecoglossus altivelis*). Mol. Biol. Rep..

[64] Biales A.D., Bencic D.C., Villeneuve D.L., Ankley G.T., Lattier D.L. (2011). Proteomic analysis of zebrafish brain tissue following exposure to the pesticide prochloraz. Aquat. Toxicol..

[65] Lu Z., Wang S., Ji C., Li F., Cong M., Shan X., Wu H. (2020). iTRAQ-based proteomic analysis on the mitochondrial responses in gill tissues of juvenile olive flounder *Paralichthys olivaceus* exposed to cadmium. Environ. Pollut..

[66] Pacitti D., Lawan M.M., Sweetman J., Martin S.A.M., Feldmann J., Secombes C.J. (2015). Selenium Supplementation in fish: A combined chemical and biomolecular study to understand Sel-Plex assimilation and impact on selenoproteome expression in rainbow trout (*Oncorhynchus mykiss*). PLoS ONE.

[67] Ferain A., Bonnineau C., Neefs I., De Saeyer N., Lemaire B., Cornet V., Larondelle Y., De Schamphelaere K.A.C., Debier C., Rees J.-F. (2018). Exploring the interactions between polyunsaturated fatty acids and cadmium in rainbow trout liver cells: A genetic and proteomic study. Aquat. Toxicol..

[68] Dang T.T., Jessen F., Martens H.J., Gringer N., Olsen K., Bøknæs N., Orlien V. (2019). Proteomic and microscopic approaches in understanding mechanisms of shell-loosening of shrimp (*Pandalus borealis*) induced by high pressure and protease. Food Chem..

[69] Green D.S., Colgan T.J., Thompson R.C., Carolan J.C. (2019). Exposure to microplastics reduces attachment strength and alters the haemolymph proteome of blue mussels (*Mytilus edulis*). Environ. Pollut..

[70] Sussarellu R., Suquet M., Thomas Y., Lambert C., Fabioux C., Pernet M.E.J., Goïc N.L., Quillien V., Mingant C., Epelboin Y. (2016). Oyster reproduction is affected by exposure to polystyrene microplastics. Proc. Natl. Acad. Sci. USA.

[71] Zhang C., Jeong C.B., Lee J.S., Wang D., Wang M. (2019). Transgenerational proteome plasticity in resilience of a marine copepod in response to environmentally relevant concentrations of cicroplastics. Environ. Sci. Technol..

[72] Magni S., Della Torre C., Garrone G., D’Amato A., Parenti C.C., Binelli A. (2019). First evidence of protein modulation by polystyrene microplastics in a freshwater biological model. Environ. Pollut..

[73] Careche M., Sánchez-Alonso I., Borda D., Nicolau A., Raspor P. (2017). Quality and quality changes assessment of processed fish. Trends in Fish Processing Technologies.

[74] Sánchez-Valencia J., Sánchez-Alonso I., Martinez I., Careche M. (2015). Low-field nuclear magnetic resonance of proton (^1^H LF NMR) relaxometry for monitoring the time and temperature history of frozen hake (*Merluccius merluccius* L.) muscle. Food Bioprocess Technol..

[75] Karoui R., Lefur B., Grondin C., Thomas E., Demeulemester C., De Baerdemaeker J., Guillard A.S. (2007). Mid-infrared spectroscopy as a new tool for the evaluation of fish freshness. Int. J. Food Sci. Technol..

[76] Hassoun A., Karoui R. (2017). Quality evaluation of fish and other seafood by traditional and nondestructive instrumental methods: Advantages and limitations. Crit. Rev. Food Sci. Nutr..

[77] Sánchez-Alonso I., Moreno P., Careche M. Low field nuclear magnetic resonance (LF NMR) spectroscopic analysis of hake (*Merluccius merluccius,* L.) upon freezing. A possibility for authentication of fresh *vs* thawed muscle. Proceedings of the 4th Trans-Atlantic Fisheries Technology Conference (TAFT).

[78] Herrero A.M., Carmona P., García M.L., Solas M.T., Careche M. (2005). Ultrastructural changes and structure and mobility of myowater in frozen-stored hake (*Merluccius merluccius* L.) muscle: Relationship with functionality and texture. J. Agric. Food Chem..

[79] Careche M., Herrero A.M., Rodriguez-Casado A., Del Mazo M.L., Carmona P. (1999). Structural changes of hake (*Merluccius merluccius* L.) fillets: Effects of freezing and frozen storage. J. Agric. Food Chem..

[80] Careche M., Sánchez-Alonso I., González-Muñoz I., Navas A., Tejada M. LF NMR relaxometry can be used to verify that fish have been subjected to freezing in order to comply with EU regulation about prevention of parasite infection. Proceedings of the 46th WEFTA Meeting.

[81] Felberg H.S., Hagen L., Slupphaug G., Batista I., Nunes M.L., Olsen R.L., Martinez I. (2010). Partial characterisation of gelatinolytic activities in herring (*Clupea harengus*) and sardine (*Sardina pilchardus*) possibly involved in *post-mortem* autolysis of ventral muscle. Food Chem..

[82] Felberg H.S., Slizyté R., Mozuraityte R., Dahle S.W., Olsen R.L., Martinez I. (2009). Proteolytic activities of ventral muscle and intestinal content of North Sea herring (*Clupea harengus*) with full and emptied stomachs. Food Chem..

[83] Skog T.-E., Hylland K., Torstensen B.E., Berntssen M.H.G. (2003). Salmon farming affects the fatty acid composition and taste of wild saithe *Pollachius virens* L.. Aquac. Res..

[84] Simmons D.B.D., Miller J., Clarence S., McCallum E.S., Balshine S., Chandramouli B., Cosgrove J., Sherry J.P. (2017). Altered expression of metabolites and proteins in wild and caged fish exposed to wastewater effluents in situ. Sci. Rep..

[85] Li W., Yao Z., Sun L., Hu W., Cao J., Lin W., Lin X. (2016). Proteomics analysis reveals a potential antibiotic cocktail therapy strategy for *Aeromonas hydrophila* infection in biofilm. J. Proteome Res..

[86] Parolini M., Bini L., Magni S., Rizzo A., Ghilardi A., Landi C., Armini A., Del Giacco L., Binelli A. (2018). Exposure to cocaine and its main metabolites altered the protein profile of zebrafish embryos. Environ. Pollut..

[87] Hartmann N.B., Hüffer T., Thompson R.C., Hassellöv M., Verschoor A., Daugaard A.E., Rist S., Karlsson T., Brennholt N., Cole M. (2019). Are we speaking the same language? Recommendations for a definition and categorization framework for plastic debris. Environ. Sci. Technol..

[88] SAPEA (2019). A Scientific Perspective on Microplastics in Nature and Society. Science Advice for Policy by European Academies—SAPEA.

[89] Lusher A., Hollman P., Mandoza-Hill J. (2017). Microplastics in Fisheries and Aquaculture.

[90] Musatadi M., González-Gaya B., Irazola M., Prieto A., Etxebarria N., Olivares M., Zuloaga O. (2020). Focused ultrasound-based extraction for target analysis and suspect screening of organic xenobiotics in fish muscle. Sci. Total Environ..

[91] Olaniyan L.W.B., Mkwetshana N., Okoh A.I. (2016). Triclosan in water, implications for human and environmental health. Springerplus.

[92] Agüera A., Fernández-Alba A.R., Piedra L., Mézcua M., Gómez M.J. (2003). Evaluation of triclosan and biphenylol in marine sediments and urban wastewaters by pressurized liquid extraction and solid phase extraction followed by gas chromatography mass spectrometry and liquid chromatography mass spectrometry. Anal. Chim. Acta.

[93] Lu J., Wang Y., Li J., Mao L., Nguyen S.H., Duarte T., Coin L., Bond P., Yuan Z., Guo J. (2018). Triclosan at environmentally relevant concentrations promotes horizontal transfer of multidrug resistance genes within and across bacterial genera. Environ. Int..

[94] Lu J., Wang Y., Zhang S., Bond P., Yuan Z., Guo J. (2020). Triclosan at environmental concentrations can enhance the spread of extracellular antibiotic resistance genes through transformation. Sci. Total Environ..

[95] Riva C., Cristoni S., Binelli A. (2012). Effects of triclosan in the freshwater mussel *Dreissena polymorpha*: A proteomic investigation. Aquat. Toxicol..

[96] Falisse E., Voisin A.S., Silvestre F. (2017). Impacts of triclosan exposure on zebrafish early-life stage: Toxicity and acclimation mechanisms. Aquat. Toxicol..

[97] Küster A., Adler N. (2014). Pharmaceuticals in the environment: Scientific evidence of risks and its regulation. Philos. Trans. R. Soc. B Biol. Sci..

[98] Zuccato E., Chiabrando C., Castiglioni S., Bagnati R., Fanelli R. (2008). Estimating community drug abuse by wastewater analysis. Environ. Health Perspect..

[99] European Monitoring Centre for Drug Addition (2020). Perspectives on Drugs. Update—Wastewater Analysis and Drugs: A European Multi-City Study. https://www.emcdda.europa.eu/system/files/publications/2757/POD_Wastewateranalysis_update2020.pdf.

[100] Brodin T., Fick J., Jonsson M., Klaminder J. (2013). Dilute concentrations of a psychiatric drug alter behavior of fish from natural populations. Science.

[101] European Union (2010). Directive 2000/60/EC of the European Parliament and of the Council of 23 October 2000 establishing a framework for Community action in the field of water policy. Off. J. Eur. Communities.

[102] European Union (2008). Directive 2008/56/EC of the European Parliament and of the Council of 17 June 2008 establishing a framework for community action in the field of marine environmental policy (Marine Strategy Framework Directive). Off. J. Eur. Communities.

[103] Martin J.M., Saaristo M., Tan H., Bertram M.G., Nagarajan-Radha V., Dowling D.K., Wong B.B.M. (2019). Field-realistic antidepressant exposure disrupts group foraging dynamics in mosquitofish. Biol. Lett..

[104] Hampel M., Alonso E., Aparicio I., Santos J.L., Leaver M. (2015). Hepatic proteome analysis of Atlantic salmon (*Salmo salar*) after exposure to environmental concentrations of human pharmaceuticals. Mol. Cell. Proteom..

[105] Yan S., Wang M., Liang X., Martyniuk C.J., Zha J., Wang Z. (2018). Environmentally relevant concentrations of carbamazepine induce liver histopathological changes and a gender-specific response in hepatic proteome of Chinese rare minnows (*Gobiocypris rarus*). Environ. Pollut..

[106] Ribas J.L.C., Sherry J.P., Zampronio A.R., Silva de Assis H.C., Simmons D.B.D. (2017). Inhibition of immune responses and related proteins in *Rhamdia quelen* exposed to diclofenac. Environ. Toxicol. Chem..

[107] Binelli A., Marisa I., Fedorova M., Hoffmann R., Riva C. (2013). First evidence of protein profile alteration due to the main cocaine metabolite (benzoylecgonine) in a freshwater biological model. Aquat. Toxicol..

[108] Li-Chan E., Li-Chan E., Chalmers J.M., Griffiths P.R. (2010). Introduction to Vibrational Spectroscopy in Food Science. Applications of Vibrational Spectroscopy in Food Science.

[109] Griffiths P.R., Li-Chan E., Chalmers J.M., Griffiths P.R. (2010). Introduction to the Theory and Instrumentation of Vibrational Spectroscopy. Applications of Vibrational Spectroscopy in Food Science.

[110] Lohumi S., Lee S., Lee H., Cho B.-K. (2015). A review of vibrational spectroscopic techniques for the detection of food authenticity and adulteration. Trends Food Sci. Technol..

[111] Li-Chan E. (1996). The applications of Raman spectroscopy in food science. Trends Food Sci. Technol..

[112] Li-Chan E., Chalmers J.M., Griffiths P.R. (2010). Instrumentation and Fundamental Applications. Applications of Vibrational Spectroscopy in Food Science.

[113] Li-Chan E., Chalmers J.M., Griffiths P.R. (2010). Analysis of Food Drink and Related Materials. Applications of Vibrational Spectroscopy in Food Science.

[114] Uddin M., Okazaki E., Li-Chan E., Chalmers J.M., Griffiths P.R. (2010). Applications of Vibrational Spectroscopy to the Analysis of Fish and Other Aquatic Food Products. Applications of Vibrational Spectroscopy in Food Science.

[115] Carmona P., Sánchez-Alonso I., Careche M., Li-Chan E., Chalmers J.M., Griffiths P.R. (2010). Chemical changes during freezing and frozen storage of fish investigated by vibrational spectroscopy. Applications of Vibrational Spectroscopy in Food Science.

[116] Gudjónsdóttir M., Arason S., Rustad T. (2011). The effects of pre-salting methods on water distribution and protein denaturation of dry salted and rehydrated cod—A low-field NMR study. J. Food Eng..

[117] Erikson U., Standal I.B., Aursand I.G., Veliyulin E., Aursand M. (2012). Use of NMR in fish processing optimization: A review of recent progress. Magn. Reson. Chem..

[118] Fan K., Zhang M. (2019). Recent developments in the food quality detected by non-invasive nuclear magnetic resonance technology. Crit. Rev. Food Sci. Nutr..

[119] Hills B.P., Takacs S.F., Belton P.S. (1989). The effects of proteins on the proton N.M.R. transverse relaxation time of water. Mol. Phys..

[120] Belton P. (2011). Spectroscopic approaches to the understanding of water in foods. Food Rev. Int..

[121] Duflot M., Sánchez-Alonso I., Duflos G., Careche M. (2019). LF ^1^H NMR T_2_ relaxation rate as affected by water addition, NaCl and pH in fresh, frozen and cooked minced hake. Food Chem..

[122] Grujić R., Petrović L., Pikula B., Amidžić L. (1993). Definition of the optimum freezing rate.1. Investigation of structure and ultrastructure of beef *M. longissimus dorsi* frozen at different freezing rates. Meat Sci..

[123] Becker B.R., Fricke B.A., Caballero B. (2003). Freezing. Principles. Encyclopedia of Food Sciences and Nutrition.

[124] Howgate P., Vaughan J.G. (1979). Fish. Food Microscopy.

[125] Shenouda S.Y.K. (1980). Theories of protein denaturation during frozen storage of fish flesh. Adv. Food Res..

[126] Haard N.F., Bligh E.G. (1992). Biochemical reactions in fish muscle during frozen storage. Seafood Science and Technology.

[127] Sánchez-Alonso I., Carmona P., Careche M. (2012). Vibrational spectroscopic analysis of hake (*Merluccius merluccius* L.) lipids during frozen storage. Food Chem..

[128] Herrero A.M., Carmona P., Careche M. (2004). Raman Spectroscopic study of structural changes in hake (*Merluccius merluccius* L.) muscle proteins during frozen storage. J. Agric. Food Chem..

[129] Lambelet P., Renevey F., Kaabi C., Raemy A. (1995). Low-field nuclear magnetic resonance relaxation study of stored or processed cod. J. Agric. Food Chem..

[130] Nott K.P., Evans S.D., Hall L.D. (1999). Quantitative magnetic resonance imaging of fresh and frozen thawed trout. Magn. Reson. Imaging.

[131] Sánchez-Alonso I., Moreno P., Careche M. (2014). Low field nuclear magnetic resonance (LF-NMR) relaxometry in hake (Merluccius merluccius, L.) muscle after different freezing and storage conditions. Food Chem..

[132] Gudjónsdóttir M., Karlsdóttir M.G., Arason S., Rustad T. (2013). Injection of fish protein solutions of fresh saithe (Pollachius virens) fillets studied by low field nuclear magnetic resonance and physicochemical measurements. J. Food Sci. Technol.-Mysore..

[133] Yano S., Tanaka M., Suzuki N., Kanzaki Y. (2002). Texture change of beef and salmon meats caused by refrigeration and use of pulse NMR as an index of taste. Food Sci. Technol. Res..

[134] Aursand I.G., Veliyulin E., Bocker U., Ofstad R., Rustad T., Erikson U. (2009). Water and salt distribution in Atlantic salmon (Salmo salar) studied by low-field 1H NMR, 1H and 23Na MRI and light microscopy: Effects of raw material quality and brine salting. J. Agric. Food Chem..

[135] Nikoo M., Regenstein J.M., Ghomi M.R., Benjakul S., Yang N., Xu X. (2015). Study of the combined effects of a gelatin-derived cryoprotective peptide and a non-peptide antioxidant in a fish mince model system. LWT Food Sci. Technol..

[136] Steen C., Lambelet P. (1997). Texture changes in frozen cod mince measured by low-field nuclear magnetic resonance spectroscopy. J. Sci. Food Agric..

[137] Jensen K.N., Guldager H.S., Jørgensen B.M. (2002). Three-way modelling of NMR relaxation profiles from thawed cod muscle. J. Aquat. Food Prod. Technol..

[138] Burgaard M.G., Jørgensen B.M. (2010). Effect of temperature on quality-related changes in cod (Gadus morhua) during short- and long-term frozen storage. J. Aquat. Food Prod. Technol..

[139] Sánchez-Alonso I., Martinez I., Sánchez-Valencia J., Careche M. (2012). Estimation of freezing storage time and quality changes in hake (Merluccius merluccius, L.) by low field NMR. Food Chem..

[140] Fu B., Labuza T.P. (1993). Shelf-life prediction: Theory and application. Food Control..

[141] Taoukis P.S., Labuza T.P., Saguy I.S., Valentas K.J., Rotstein E., Singh R.D. (1997). Kinetics of food deterioration and shelf-life prediction. The Handbook of Food Engineering Practice.

[142] Careche M., Sánchez-alonso I., Martinez I., Webb P.G.A. (2018). Estimation of quality in frozen fish by low field NMR. Modern Magnetic Resonance.

[143] Sánchez-Valencia J., Sánchez-Alonso I., Martinez I., Careche M. (2014). Estimation of frozen storage time or temperature by kinetic modeling of the Kramer shear resistance and water holding capacity (WHC) of hake (*Merluccius merluccius*, L.) muscle. J. Food Eng..

[144] Careche M., Carmona P., Sánchez-Alonso I. (2015). Monitoring the time and temperature history of frozen hake (*Merluccius merluccius*, L.) muscle by FTIR spectroscopy of the lipid fraction. Food Bioprocess Technol..

[145] Velioglu H.M., Temiz H.T., Boyaci I.H. (2015). Differentiation of fresh and frozen-thawed fish samples using Raman spectroscopy coupled with chemometric analysis. Food Chem..

[146] EU Commission (2011). Regulation (EU) No 1276/2011 of 8 December 2011. Off. J. Eur. Union.

